# Balance circuit classes to improve balance among rehabilitation inpatients: a protocol for a randomised controlled trial

**DOI:** 10.1186/1471-2318-13-75

**Published:** 2013-07-20

**Authors:** Daniel Treacy, Karl Schurr, Catherine Sherrington

**Affiliations:** 1 Musculoskeletal Division, The George Institute for Global Health, The University of Sydney, Missenden Rd, PO Box M201, Sydney, Australia; 2 Bankstown-Lidcombe Hospital, PO Box: Locked Mail bag 1600, Bankstown, NSW, 2200, Australia

**Keywords:** Balance, Rehabilitation, Inpatients, Circuit classes, Physiotherapy, Exercises

## Abstract

**Background:**

Impaired balance and mobility are common among rehabilitation inpatients. Poor balance and mobility lead to an increased risk of falling. Specific balance exercise has been shown to improve balance and reduce falls within the community setting. However few studies have measured the effects of balance exercises on balance within the inpatient setting.

The aim of this randomised controlled trial is to investigate whether the addition of circuit classes targeting balance to usual therapy lead to greater improvements in balance among rehabilitation inpatients than usual therapy alone.

**Methods/Design:**

A single centre, randomised controlled trial with concealed allocation, assessor blinding and intention-to-treat analysis. One hundred and sixty two patients admitted to the general rehabilitation ward at Bankstown-Lidcombe Hospital will be recruited. Eligible participants will have no medical contraindications to exercise and will be able to: fully weight bear; stand unaided independently for at least 30 seconds; and participate in group therapy sessions with minimal supervision.

Participants will be randomly allocated to an intervention group or usual-care control group. Both groups will receive standard rehabilitation intervention that includes physiotherapy mobility training and exercise for at least two hours on each week day. The intervention group will also receive six 1-hour circuit classes of supervised balance exercises designed to maximise the ability to make postural adjustments in standing, stepping and walking.

The primary outcome is balance. Balance will be assessed by measuring the total time the participant can stand unsupported in five different positions; feet apart, feet together, semi-tandem, tandem and single-leg-stance. Secondary outcomes include mobility, self reported physical functioning, falls and hospital readmissions. Performance on the outcome measures will be assessed before randomisation and at two-weeks and three-months after randomisation by physiotherapists unaware of intervention group allocation.

**Discussion:**

This study will determine the impact of additional balance circuit classes on balance among rehabilitation inpatients. The results will provide essential information to guide evidence-based physiotherapy at the study site as well as across other rehabilitation inpatient settings.

**Trial registration:**

The protocol for this study is registered with the Australian New Zealand, Clinical Trials Registry: ACTRN=12611000412932

## Background

Patients admitted to a general rehabilitation ward often present with poor mobility, impaired balance and reduced ability to carry out activities of daily living [1,2]. These impairments usually result from a primary diagnosis of fall, orthopaedic complaint (commonly hip fracture), neurologic condition or frailty.

Balance is defined as the ability to maintain the projection of the body's centre of mass within manageable limits of the base of support [3]. Poor balance and mobility impairment have consistently been associated with an increased risk of falling among rehabilitation inpatients and among patients discharged home from a rehabilitation setting [4-8].

Falls are a frequent occurrence among patients admitted to a rehabilitation ward [9,10] and once discharged home the likelihood of falling is significantly greater for these people than that for the general community [11,12]. Falls are the leading cause of injury related hospitalisations in NSW, accounting for 39% of all such hospitalisations in NSW in the period 2005/06 to 2007/08. Almost two thirds of hospital admissions for people aged 65 and over are falls related. In 2006/07 this equated to 49,485 hospitalisations. Total health care costs in NSW associated with fall injuries in 2006/07 was estimated at $558.5 million [13], more than any other single cause of injury. In addition to the financial cost, falls also place significant burden on an individual’s quality of life.

Specific balance exercise has been shown to improve balance and reduce falls in the general older population. There have been two systematic reviews looking at exercise interventions aimed at improving balance [14] or decreasing falls [15] in older people. Howe et al. [14] found that programmes that involved balance and coordination were effective interventions for improving balance. Sherrington et al. [15] found that exercise programmes reduced falls and programmes that specifically included challenging balance activities such as exercising without using the hands for support and narrowing the base of support were associated with a greater fall prevention effect. This is consistent with a “task-specific” approach to exercise prescription i.e., that greater improvements are seen when exercises are most similar to the task for which improvement is sought [16].

Few studies have measured the effects of balance exercises on balance within the inpatient setting. While Sherrington et al. [17] found that balance significantly improved after 2 weeks of either weight-bearing balance exercise or a non-weight bearing strengthening program for inpatients with hip fracture, no difference was found between the two exercise groups. Haines et al. [10] reported a reduction in incidence of falls in the rehabilitation inpatient setting after a targeted falls prevention programme incorporating individualised balance exercises. However, another targeted multifactorial intervention incorporating balance exercise showed no effect on incidence rate of falls compared with usual care [18]. Balance was not assessed/measured by either Haines et al. [10] or Cumming et al. [18].

The exercise principles found to be associated with a greater impact on falls in the Sherrington et al. review [15] (i.e., exercising without using the hands for support and narrowing the base of support to challenge postural adjustments in standing) will be implemented in this trial to determine whether similar improvements in balance can be made among patients undertaking general rehabilitation. To our knowledge this will be the first study to examine the effects of a balance exercise programme on balance among patients admitted to a general rehabilitation ward. The primary research question is:

Does the addition of balance circuit classes to usual therapy lead to greater improvements in balance among rehabilitation inpatients than usual therapy?

## Methods/Design

### Design

**Figure 1:**
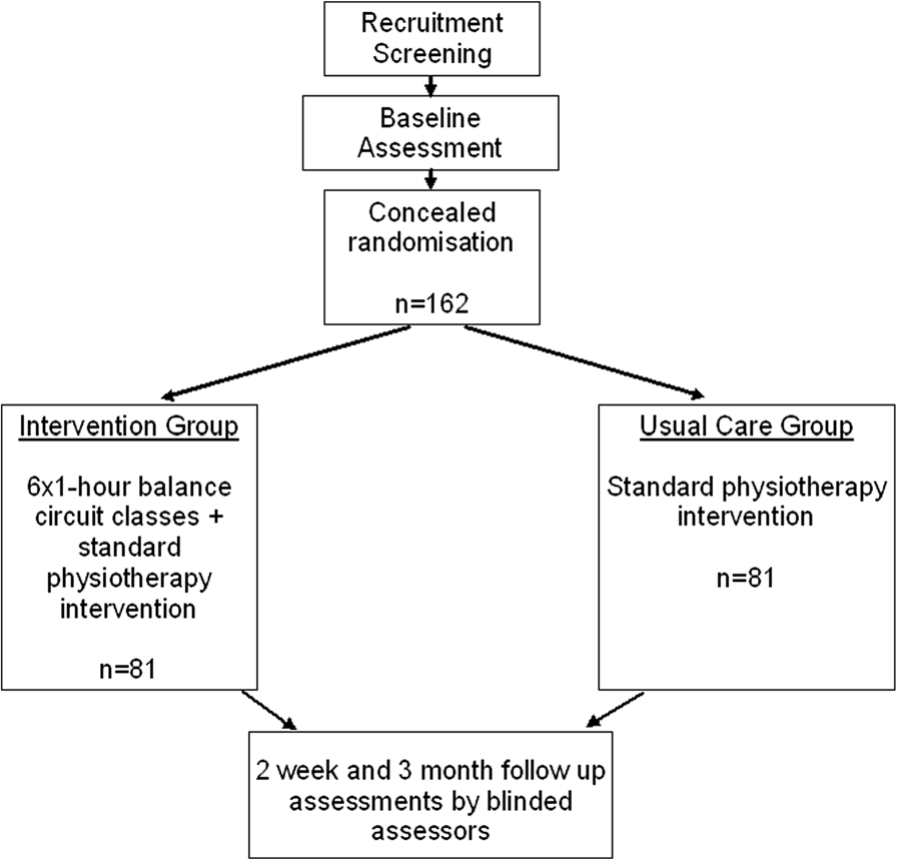
Flow of participants.

A single centre, randomised controlled trial with concealed allocation, assessor blinding and intention to treat analysis with three month follow up will be conducted among 162 rehabilitation inpatients. Figure 1 gives an overview of the study design.

### Participants

All patients admitted to the adult general rehabilitation ward at Bankstown-Lidcombe Hospital will be screened for study eligibility. Patients will be eligible if they are: admitted to the ward for rehabilitation; are able to stand for 30 seconds without physical assistance or the help of an assistive device; have no contraindications to exercise, such as uncontrolled hypertension or unstable cardiac disease; are able to fully weight bear as ordered by a medical officer; and are suitable for a group exercise class with minimal supervision as determined by the treating physiotherapist. Patients with a known multi-resistant organism infection or other infection that would pose a significant risk to others in a group setting (e.g. Methicillin-Resistant Staphylococcus Aureus, Vancomycin Resistant Enterococci, Tuberculosis) will be excluded.

Patients with a Mini Mental State Examination (MMSE) score of more than 17 will be asked to give informed consent to study participation. Consent will be sought from a ‘person responsible’ for patients who score 17 or less on the MMSE as well as for individuals who score more than 17 but treating staff consider to have a cognitive impairment that would hamper the ability to give informed consent. Interpreters will be used for consent and study assessments as required.

The South West Sydney Local Health District Human Research Ethics Committee (HREC) approved the study protocol (HREC/10/LPOOL/187).

### Randomisation

A concealed allocation procedure (numbered sealed opaque envelopes) will be used to randomly assign participants to intervention or usual-care control groups after consent and baseline measurements are performed. The allocation schedule will be computer generated using randomly ordered blocks of four and six. The schedule and sealed opaque envelopes will be prepared by an allied health staff member at the study site not involved in study recruitment or intervention.

### Intervention

All participants will receive usual therapy consisting of assessment and treatment by the multidisciplinary ward team at Bankstown-Lidcombe Hospital. Patients are predominately treated within a group setting in physiotherapy with additional one to one sessions as required with the focus being on weight bearing exercises. Patients are seen once or twice per day and on most would spend at least two hours per day in physiotherapy. The majority of physiotherapy practice occurs within the rehabilitation gym. Physiotherapy staff members are not rostered to work on weekends or public holidays. Staffing consists of two full time physiotherapists and a half-time physiotherapy assistant for twenty beds.

The intervention group will also receive six 1-hour standing balance circuit classes over a two-week period. Sessions will be run each Monday, Wednesday and Friday unless physiotherapy staff members are absent due to a public holiday. Following the two-week period, participants in the intervention group will be invited to continue with the balance circuit classes until discharged from hospital. Participants in both groups will receive usual multidisciplinary team care (e.g. outpatient therapy) after discharge. This may include outpatient physiotherapy performed either within the hospital in an outpatient setting or in the patient’s home. Physiotherapy intervention as an outpatient would generally be twice a week for six weeks.

The standing balance circuit class will comprise seven stations, each station consisting of a different exercise. The class will be supervised by one to two physiotherapy staff members (this may include a Physiotherapy Assistant) with a maximum of eight participants. The staff members running the class will be encouraged to increase the difficulty of the exercise depending on the ability of individual patients. Class participants will spend six minutes at each exercise station and will use six of the seven stations during each session. All stations have been designed to challenge postural adjustments while standing and stepping. This challenge will be achieved by performing exercises without the use of hands for support and by narrowing the base of support as able. Participants will be progressed to more challenging balance exercises as deemed appropriate by the treating physiotherapist. The amount, or dosage of exercises completed at each station will be recorded. The seven balance stations are outlined in Table 1.

**Table 1:** Balance stations

**Station**	**Exercise**	**Progression**	**Counting/recording**
**Catching and passing**	Patient to catch and pass a ball with the therapist or another patient	To increase the difficulty of this task the ball can be passed to a distance further than arm’s reach. The task difficulty may also be increased by the patient decreasing their centre of balance by standing with their feet closer together or stand on an unstable surface such as thick foam rubber.	Each attempted catch to be recorded as one repetition.
**Stepping forward**	Patient to step forward with one leg then step back, continuing with the alternate leg.	To increase the difficulty of the task a block can be used to step up onto.	Every step forward and back to be recorded as one repetition
**Sideways stepping**	Patient to step sideways.	To increase difficulty of the task the patient can increase their step length or step over an object.	Each step sideways to be recorded as one repetition.
**Stepping grid**	Patient to step to targets on a board. There should be four targets (one to the right side, one to the left side, one in front of the right foot and one in front of the left foot	To increase difficulty of the task the targets can be moved to a distance further away from the patient. Additional targets may also be placed behind the person to increase the difficulty	Each step is recorded as one repetition
**Weight shift forwards and backwards**	Patient to shift weight forwards and backwards using a sway meter [19]. Technology such as the Wii platform may be used instead of the Sway meter	To increase the difficulty of the task the patient can follow a shape in various directions	Each full movement is recorded as one repetition
**Heel raises**	Patient to stand on toes, then lower self down till the feet are flat	To increase the difficulty of the task the patient can perform the heel raises on the edge of a block or on one leg.	Each heel raise is recorded as one repetition
**Reaching and moving objects**	Patient to reach for an object on one side of their body than move the object to the other side using the same arm	To increase the difficulty of the task the patient may decrease their centre of balance by standing with their feet closer together or stand on an unstable surface	Each movement of the object is recorded as one repetition.

### Outcome measures

Demographic data will be collected by baseline interview and from the medical record and will include age, sex, presenting diagnosis, usual pre-morbid type of residence, discharge location, pre-morbid level of function (whether the participant could climb a flight of stairs and walk 800 m in the three months prior to hospital admission), 12 month fall history, rehabilitation length of stay and total hospital length of stay.

#### Primary outcome

The primary outcome measures will be balance at two weeks and at three months after randomisation. This will be assessed using a composite balance measure with five balance tests: feet apart, feet together, semi-tandem (heel of one foot beside the big toe of the other foot), tandem and single leg stance. Each test will be performed without aid or upper limb support and timed up to a maximum of 10 seconds (range = 0 to 50 sec) with stand by assistance from the therapist.

#### Secondary outcomes

Secondary outcomes will include mobility, self reported physical functioning, fall incidence and hospital readmissions.

Mobility will be assessed using the lower extremity Summary Performance Score [20] version of the Short Physical Performance Battery [21]. This battery gives a composite score based on timed performance of three mobility tasks: the ability to stand up for 10 seconds with feet in different positions (together side by side, semi-tandem and tandem), 4-metre walk and time to rise (stand-up) from a chair five times.

Self reported physical functioning will be assessed using the Basic Mobility and Daily Activity domains of the Computer Adaptive Testing version of the Boston University Activity Measure for Post Acute Care (AM-PAC) (Haley et al. 2004) [22]. Computer Adaptive Testing uses a computer algorithm to pre-select the items that will be administered to a specific patient based on their responses to previous items. The AM-PAC measures functional outcome by using Item Response Theory (IRT). The AM-PAC Basic Mobility domain includes 101 items that address basic movement and physical functioning activities, such as bending, walking, carrying and climbing stairs. The AM-PAC Daily Activity domain includes 70 items that address basic self-care and instrumental activities of daily life.

Information about falls and hospital readmissions will be collected via interview at two-weeks and at three-months and confirmed via hospital records data.

### Data collection

Balance and mobility will be assessed at baseline, two weeks and at three months after randomisation. Self reported physical functioning will be assessed at two weeks and at three months after randomisation. Information about falls and hospital readmissions will be collected at two weeks and at three months after randomisation. A proxy will be used for assessment of self reported physical functioning and fall/hospital readmission data if the assessor had significant concern over the reliability of information collected from the participant. Blinded assessors (experienced therapists) will conduct follow-up assessments.

Pre-morbid level of functioning (whether the participant could climb a flight of stairs and walk 800 m in the three months prior to hospital admission) and 12-month fall history will be collected during the baseline assessment. Medical records will be extracted to determine presenting diagnosis and demographic data.

### Sample size calculation

A sample of 162 participants will be required to detect a between-group difference of 3 secs for the five balance tests (assuming a standard deviation of 9, power = 80%, p = 0.05, correlation between baseline and follow-up measures 0.65 and 15% drop out rate). Data from a previous study [4] indicate that 3 secs is likely to be approximately 15% of discharge values and we consider an effect of this size to be a clinically worthwhile.

### Statistical analysis

Between group differences in the primary outcome and secondary measures at the two week and three month follow-ups will be analysed using ANCOVA models where baseline values are entered as covariates. Dichotomous outcomes will be analysed via logistic regression models. Primary analyses will use an intention-to-treat approach. A secondary per protocol analysis will be undertaken using data from those who participated in at least three of the six intervention sessions.

## Discussion

This trial will provide essential information to guide evidence-based physiotherapy at the study site as well as across other rehabilitation inpatient settings. Specifically, it will identify whether the addition of a two-week circuit-class balance program improves balance, mobility and function among rehabilitation inpatients. Decreased balance and its subsequent effects such as falls decreased mobility and functional within the inpatient setting is a significant and costly issue for the health system. Despite this cost there has been very little research to date examining the effectiveness of exercise programs designed to improve balance in this setting.

The exercise approach in this standing balance circuit class have been designed using evidence from systematic reviews and randomised trials in other settings in which they have been demonstrated to improve balance and decrease falls. The ratio of up to 8 patients for two therapists ensures that it is a cost-efficient program and feasible in a rehabilitation setting. The eligibility criteria for the circuit class have been designed to ensure that the program is appropriate for a broad range of rehabilitation inpatients. The two-week intervention period, though short, has been designed to fit in with usual practice.

If shown to be effective, this balance program has the potential to improve the balance, mobility and function of future rehabilitation inpatients both at the study site and across other similar settings where this program is implemented. These physical function improvements have potential to transfer to an improved quality of life for patients and their families and carers. In addition, gains in balance and function may also reduce fall rates, which would confer significant benefit to the wider community in terms of reducing the financial burden associated with falls.

## Competing interests

The authors declare that they have no competing interests.

## Authors’ contributions

All authors drafted the manuscript. All authors are actively involved in the study. All authors read and approved the final manuscript.

## Pre-publication history

The pre-publication history for this paper can be accessed here:

http://www.biomedcentral.com/1471-2318/13/75/prepub
